# Longitudinal associations between adolescents’ individualised risk for depression and inflammation in a UK cohort study

**DOI:** 10.1016/j.bbi.2021.12.027

**Published:** 2022-03

**Authors:** Rachel M. Latham, Christian Kieling, Louise Arseneault, Brandon A. Kohrt, Terrie E. Moffitt, Line J.H. Rasmussen, Thiago Botter-Maio Rocha, Valeria Mondelli, Helen L. Fisher

**Affiliations:** a King’s College London, Social, Genetic and Developmental Psychiatry Centre, Institute of Psychiatry, Psychology and Neuroscience, London, UK; b ESRC Centre for Society and Mental Health, King’s College London, London, UK; c Department of Psychiatry, Universidade Federal Do Rio Grande Do Sul, Porto Alegre, Brazil; d Child and Adolescent Psychiatry Division, Hospital de Clínicas de Porto Alegre, Porto Alegre, Brazil; e Division of Global Mental Health, George Washington University, Washington, DC, USA; f Department of Psychology and Neuroscience, Duke University, Durham, NC, USA; g Psychiatry and Behavioral Sciences, Duke University, Durham, NC, USA; h Center for Genomic and Computational Biology, Duke University, Durham, NC, USA; i PROMENTA, Department of Psychology, University of Oslo, Norway; j Department of Clinical Research, Copenhagen University Hospital Amager and Hvidovre, Hvidovre, Denmark; k King’s College London, Department of Psychological Medicine, Institute of Psychiatry, Psychology and Neuroscience, King’s College London, London, UK; l National Institute for Health Research Mental Health Biomedical Research Centre, South London and Maudsley NHS Foundation Trust and King's College London, London, UK

**Keywords:** Adversity, Adolescence, Biomarkers, Major depressive disorder, Mental health, Psychopathology, Prevention, Risk factors, Transition to adulthood

## Abstract

Inflammation is associated with poor physical and mental health including major depressive disorder (MDD). Moreover, there is evidence that childhood adversity – a risk factor for MDD – becomes biologically embedded via elevated inflammation. However, the risk of developing MDD arises from multiple sources and yet there has been little investigation of the links between individuals’ constellation of MDD risk and subsequent inflammation. We therefore examined associations between individual risk for MDD calculated in early adolescence and levels of inflammation six years later. We use data from the Environmental Risk (E-Risk) Longitudinal Twin Study, a nationally representative UK birth cohort of 2,232 children followed to age 18 with 93% retention. Participants’ individual risk for developing future MDD was calculated at age 12 using a recently developed prediction model comprising multiple psychosocial factors. Plasma levels of three inflammation biomarkers were measured at age 18: C-reactive protein (CRP), interleukin-6 (IL-6), and a newer biomarker, soluble urokinase plasminogen activator receptor (suPAR), which is thought to reflect the level of systemic chronic inflammation. MDD risk scores calculated at age 12 were positively associated with levels of suPAR (but not CRP or IL-6) at age 18 after adjusting for key covariates (b = 1.70, 95% CI = 0.46 – 2.95, p = 0.007). Adolescents at high risk of MDD (risk scores ≥ 90th centile) had significantly higher mean levels of suPAR six years later than adolescents who had been identified as low risk (risk scores ≤ 10th centile) (b = 0.41, 95% CI = 0.18 – 0.64, p < 0.001). Findings support the notion that childhood psychosocial risk for MDD leads to increased levels of low-grade inflammation. If replicated in studies with repeated assessments of inflammation biomarkers throughout childhood and adolescence, these findings would support targeted interventions to reduce inflammation, as measured by suPAR, for adolescents at high risk of MDD to potentially prevent development of depression and physical health problems related to chronic inflammation.

## Highlights

• Risk of future MDD and inflammation biomarkers were measured in a UK birth cohort.
• Adolescents’ risk for MDD positively associated with levels of suPAR 6 years later.
• Those at high MDD risk subsequently had higher suPAR compared to those at low risk.
• However, no associations were found between MDD risk and levels of CRP or IL-6.
• MDD risk arising from multiple sources may become biologically embedded over time.

## 1 Introduction

Inflammation is part of the body’s innate immune response to protect itself from harmful stimuli. Although typically thought of as a response to physical threats, there is substantial evidence that psychological stressors can trigger an immune response too (Baumeister et al., 2016, Glaser and Kiecolt-Glaser, 2005) particularly those that involve cues for physical or social threat such as conflict, evaluation, rejection, and isolation (Slavich and Cole, 2013). A timely inflammatory response is a critical defence mechanism against disease and illness. However prolonged, systemic inflammation (over months or years) can harm physical health (e.g., cardiovascular disease) and evidence suggests it is also associated with poor mental health including major depressive disorder (MDD), a debilitating mental illness that commonly begins in adolescence and young adulthood.

The causal order of associations between inflammation and MDD are unknown and still controversial. For example, cross-sectional observational studies have shown small, elevated levels of circulating inflammation biomarkers (such as interleukin-6 (IL-6) and C-reactive protein (CRP)) among depressed individuals in community and clinical samples (Howren et al., 2009). Furthermore, longitudinal population-based studies have found higher levels of IL-6 and CRP at baseline to be associated with an increased average likelihood of depression several years later (Khandaker et al., 2014, Zalli et al., 2016). However, noting that not everyone with depression has elevated levels of inflammation (Raison et al., 2006) work to better understand their co-occurrence has highlighted the role of childhood adversity. For instance, in both children and adults, current depression in combination with a history of childhood maltreatment has been found to confer significantly elevated levels of CRP whereas this elevation was not evident in depressed individuals who were not maltreated (Danese et al., 2008, Danese et al., 2011). A similar coupling of depression with elevated CRP and IL-6 has also been found among female adolescents who had experienced childhood adversity (Miller and Cole, 2012). These findings – together with a wealth of evidence demonstrating links between childhood stress and inflammation (Baldwin et al., 2018, Baumeister et al., 2016, Coelho et al., 2014, Danese et al., 2007) – suggest a biological embedding of childhood psychosocial risk that is associated with depression (Slavich and Irwin, 2014, Danese and Baldwin, 2017).

Risk factors for MDD other than childhood adversity have been little explored in relation to inflammation in adolescence and young adulthood (Zajkowska et al., 2021). Examination of this is particularly important given the common onset of MDD during this developmental period. Moreover, despite recognition that multiple risk factors will combine to increase the likelihood of someone developing depression (Gerard and Buehler, 2004, Hankin, 2012), there has been limited exploration of whether individuals’ constellation of risk is related to inflammation. The aforementioned study by Miller and Cole (2012) indexed childhood adversity by summing several components (family socioeconomic factors, parental separation, and parental affective illness), thus treating each exposure with equal weighting. A more nuanced approach and examination of a broader range of MDD risk factors in combination is needed to better understand associations between MDD risk and inflammation in young people.

To address this gap in the literature we examine whether young adolescents’ individual risk for MDD (arising from multiple psychosocial risk factors) predicts later inflammation to see if this constellation of risk becomes biologically embedded. To do this, we utilise a recently developed multivariable prognostic model (Rocha et al., 2021) that calculates individual risk in early adolescence of developing MDD at age 18, thus capturing the peak age for depression onset (Hankin et al., 1998). Rather than explaining risk for MDD at the average – or ‘group’ – level as much prior work has done, this model uses a combination of variables to predict the risk of MDD onset for a particular individual, calculated as a risk score. Originally developed using a Brazilian sample, this prediction model was then externally validated in a UK nationally representative cohort of children followed from birth to age 18, the Environmental Risk (E-Risk) Longitudinal Twin Study. The UK model used social and demographic risk factors measured at age 12 (sex, skin colour, drug use, school failure, social isolation, fight involvement, child maltreatment, history of running away from home, and interactions of each of these with sex) to predict individuals’ risk of developing MDD at age 18 and did so with reasonable accuracy (Rocha et al., 2021).

Using the individual MDD risk scores calculated by this model at age 12 we examine here associations with inflammation biomarkers measured when E-Risk study participants were aged 18, accounting for key covariates. Specifically, we separate out those individuals with high and low constellations of MDD risk at age 12 and investigate whether they subsequently differ in levels of CRP, IL-6, and soluble urokinase plasminogen activator receptor (suPAR) at age 18. In contrast to CRP and IL-6, which are affected by acute fluctuations in inflammation levels, for example due to infections (Hunter and Jones, 2015, Rhodes et al., 2011), suPAR is an emerging marker of systemic chronic inflammation that is less affected by acute changes and short-term influences (Rasmussen et al., 2021a, Rasmussen et al., 2021b). Recent work has shown that suPAR was associated with adverse childhood experiences (Rasmussen et al., 2019), including in the E-Risk study sample (Rasmussen et al., 2020), and adult stressful life events (Bourassa et al., 2021), while CRP and IL-6 were not consistently associated with these stressors. Therefore, suPAR may be a useful way of capturing the longer-term inflammatory impacts of early adolescent MDD risk. We hypothesise a positive relationship between individual MDD risk scores calculated at age 12 and levels of inflammation measured at age 18 such that those with high-risk scores will have higher levels of inflammation than those with low-risk scores. By focusing on young adolescents’ risk for developing future MDD, and controlling for any previous depressive symptoms, we can examine whether the combined risk for depression arising from multiple sources becomes biologically embedded such that it is evident in inflammatory biomarkers six years later.

## 2 Methods

### 2.1 Sample

Participants were members of the Environmental Risk (E-Risk) Longitudinal Twin Study, which tracks the development of a nationally representative birth cohort of 2,232 British twin children. Full details about the sample are reported elsewhere (Moffitt and E-Risk Study Team, 2002) and in the Supplementary Material. Briefly, the E-Risk sample was constructed in 1999–2000 when 1,116 families (93% of those eligible) with same-sex 5-year-old twins participated in home-visit assessments. This sample comprised 56% monozygotic (MZ) and 44% dizygotic (DZ) twin pairs; sex was evenly distributed within zygosity (49% male). Families were recruited to represent the UK population of families with newborns in the 1990s, on the basis of residential location throughout England and Wales and mother’s age.

Follow-up home-visits were conducted when the participants were aged 7, 10, 12 and 18 years (participation rates were 98%, 96%, 96%, and 93%, respectively). There were 2,066 E-Risk participants who were assessed at age 18. There were no differences between those who did and did not take part at age 18 in terms of socioeconomic status (SES) assessed when the cohort was initially defined (χ^2^ = 0.86, *p* = 0.65), age-5 IQ scores (*t* = 0.98, *p* = 0.33), age-5 behavioural (*t* = 0.40, *p* = 0.69) or emotional (*t* = 0.41, *p* = 0.68) problems, or childhood poly-victimisation (*z* = 0.51, *p* = 0.61). The cohort’s neighbourhoods represent the full range of socioeconomic conditions in Great Britain. Supplementary Figure S1 shows E-Risk families’ addresses compared to the deciles of the UK’s 2015 Lower-layer Super Output Area (LSOA) Index of Multiple Deprivation (IMD) which averages 1,500 residents; approximately 10% of the cohort fills each of IMD’s 10% bands, a near-perfect match to the population.

The Joint South London and Maudsley and the Institute of Psychiatry Research Ethics Committee approved each phase of the study. Parents gave informed consent and twins gave assent between 5 and 12 years and then informed consent at age 18.

### 2.2 Measures

#### 2.2.1 Age-12 risk of future MDD

At age 12, individual risk scores for age-18 MDD were calculated using a multivariable prognostic model (full details of model development and validation are described by Rocha et al., 2021). Briefly, this model was initially developed using data from the 1993 Brazil Pelotas birth cohort with sociodemographic variables collected at age 15 to predict individual risk of MDD at age 18. The model was then externally validated and refitted to the UK E-Risk cohort with a reasonable level of accuracy (C-Statistic = 0.62; see Supplementary Material and Supplementary Table S1 for further details). Model predictors, measured at age 12 in E-Risk included: biological sex (male/female); skin colour (white/non-white); any drug use (yes/no); school failure (yes/no); social isolation (yes/no); fight involvement (yes/no); ever ran away from home (yes/no); childhood maltreatment (none/probable/severe); and interactions of each of these with biological sex (for measurement details see Supplementary Table S2). The outcome of interest, a major depressive disorder episode in the previous 12 months, was assessed via interview at age 18 based on DSM-IV criteria. In the E-Risk sample, 18.5% (N = 414) had a research diagnosis of DSM-IV MDD at age 18. For the current analyses, MDD risk scores were calculated for E-Risk participants who had an intelligence quotient ≥ 70, were assessed for MDD at age 18, and had data for all model predictors (N = 1,489; mean risk score = 0.18, *SD* = 0.06).

#### 2.2.2 MDD risk group membership

We categorised adolescents who were at low risk for age-18 MDD as those with risk scores equal to or below the 10th percentile (low-risk group; N = 173). Adolescents at high risk for age-18 MDD were identified as those with risk scores equal to or above the 90th percentile (high-risk group; N = 243). Percentile cut-offs were derived from the E-Risk refitted model reported by Rocha et al. (2021). Importantly, because the probability of depression is known to be higher in females than males, we generated sex-specific percentile thresholds (this follows the procedure adopted by Kieling et al., 2021). The probability of developing MDD at age 18 for the 10th and 90th percentiles were 17% and 29% respectively for females, and 12% and 16% respectively for males.

#### 2.2.3 Age-18 inflammation biomarkers

Venous blood was collected with EDTA tubes from 1,700 of the 2,066 participants (82.3%) who participated in the age-18 home visits. Tubes were spun at 2500 g for 10 min and plasma samples obtained. Samples were stored at -80 °C. Plasma CRP (high-sensitivity CRP) was measured using enzyme-linked immunosorbent assay (ELISA) (Quantikine ELISA Kit DCRP00, R&D Systems) following the manufacturer’s protocol (N = 1,448). The coefficient of variation was 5.6%. Plasma IL-6 levels were measured using ELISA (Quantikine HS ELISA Kit HS600C, R&D Systems) following the manufacturer’s protocol (N = 1,448). The coefficient of variation was 12.6%. Plasma suPAR levels were analysed using ELISA (suPARnostic AUTO Flex ELISA, ViroGates A/S) following the manufacturer’s protocol (N = 1,447). The coefficient of variation was 6%.

#### 2.2.4 Covariates

Due to their potential associations with inflammation biomarkers, we adjusted for body mass index (BMI; calculated as weight in kilograms divided by height in meters squared), body temperature (measured at the time of inflammation biomarker assessment), and current daily smoking (yes/no based on self-reported number of cigarettes per day, on average) all measured at the age-18 assessment.

Additionally, due to potential associations with MDD risk scores and inflammation, we adjusted for depression experienced by the age of 12. Childhood depressive symptoms were reported by mothers when children were aged 5, 7, and 10 using a 7-item depression subscale derived from the Child Behaviour Checklist (Achenbach, 1991) for emotional problems. Each item was coded as 0 (not true), 1 (somewhat/sometimes true), or 2 (very true or often true); items were summed and the 93rd percentile was used as a cut point. At age 12, depressive symptoms were self-reported using the Children’s Depression Inventory (Kovacs, 1992) in private interviews. Items were summed and a score equal to or greater than 20 was used as the clinical cut point. In total, 22.6% (N = 504) of E-Risk participants had experienced clinically significant depressive symptoms by age 12.

For analyses that used continuous age-12 risk scores, we also included sex as a covariate because of its association with MDD and potential association with inflammation. However, sex was not included as a covariate in the risk group analyses because we used sex-specific percentiles to categorise depression risk group membership.

### 2.3 Statistical analyses

The depression risk prediction model was run using the software R (version 3.6.3); all other analyses were conducted using Stata (version 15). E-Risk participants with inflammation levels below the assays’ detection limit or greater then 4SDs above the mean of CRP (n = 18), IL-6 (n = 8), or suPAR (n = 3) were excluded, leaving 1,430 participants (69.2% of those who took part at age 18) with CRP data, 1,440 (69.7%) with IL-6 data, and 1,444 (69.9%) with suPAR data. Both CRP and IL-6 levels were log-transformed to improve the normality of their distributions (as per Rasmussen et al., 2020). We used linear regression models to examine associations between MDD risk scores calculated at age 12 and levels of CRP, IL-6, and suPAR measured at age 18.

First, we used age-12 risk scores as a continuous variable. Models were corrected for familial clustering using the ‘CLUSTER’ command. We adjusted for sex, BMI, body temperature, smoking, and previous depressive symptoms to test the robustness of associations. Second, we examined whether MDD risk group membership (i.e., low- or high-risk) at age 12 was associated with levels of CRP, IL-6, and suPAR at age-18. As before, models were corrected for familial clustering, and then additionally adjusted for BMI, body temperature, smoking, and previous depression.

Missing data was predominantly due to participants not having inflammation biomarker data available (see Measures) therefore we analysed complete cases. MDD risk scores did not differ between those with and those without any inflammation data (*t* = -0.41, *p* = 0.679). The number of E-Risk participants with a MDD risk score calculated at age 12 and inflammation data at age 18 varied according to the specific biomarker (N = 1,026 for CRP; N = 1,034 for IL-6; and N = 1,039 for suPAR). Of these, 981 participants (for CRP), 989 (for IL-6) and 994 (for suPAR) also had complete data for all covariates and thus comprised the samples for our fully adjusted analyses.

## 3 Results

### 3.1 Descriptive statistics

**Fig. 1:**
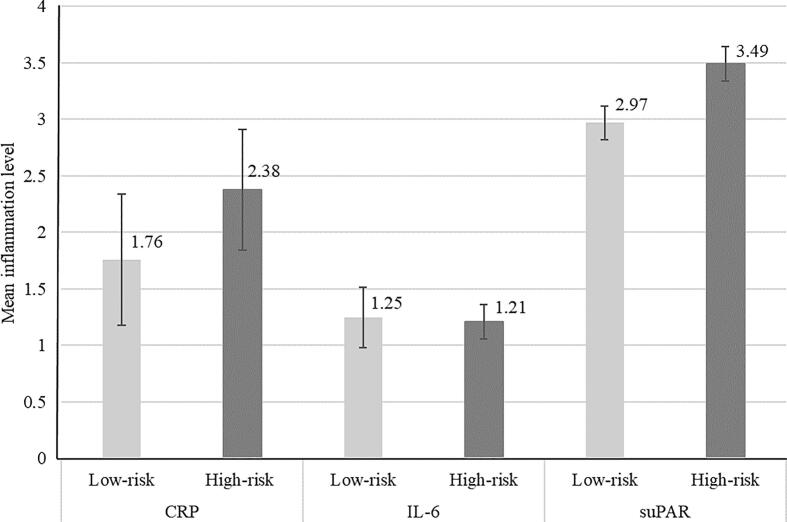
Mean levels of CRP (mg/L), IL-6 (pg/L), and suPAR (ng/mL) at age 18 according to age-12 MDD risk group membership. *Note.* CRP = C-reactive protein; IL-6 = interleukin-6; MDD = major depressive disorder; suPAR = soluble urokinase plasminogen activator receptor. Low-risk group defined as a risk score for age-18 MDD ≤ 10th percentile (N = 116 for CRP; N = 117 for both IL6 and suPAR); high-risk group defined as a risk score for age-18 MDD ≥ 90th percentile (N = 171 for CRP; N = 170 for both IL6 and suPAR). Error bars show 95% confidence intervals.

Mean levels of inflammation biomarkers at age 18 among participants with MDD risk scores were as follows: CRP mean = 2.32 mg/L (SD = 3.67), IL-6 mean = 1.22 pg/mL (SD = 1.24), and suPAR mean = 3.21 ng/mL (SD = 0.91). Fig. 1 shows the mean levels of age-18 CRP, IL-6 and suPAR according to MDD risk group membership at age 12.

### 3.2 Associations between Age-12 risk of future MDD and Age-18 inflammation biomarkers

Table 1 shows the results of the regression models predicting levels of CRP, IL-6, and suPAR from individual risk scores for MDD. Higher MDD risk scores calculated at age 12 were associated with significantly higher levels of suPAR at age 18. This association remained when models were adjusted for BMI, body temperature, smoking, sex, and previous depressive symptoms. Higher MDD risk scores were also associated with higher levels of CRP, but this association was attenuated once covariates were adjusted for. We found no significant associations between MDD risk scores and age-18 levels of IL-6.

**Table 1:** Regression Models Predicting Age-18 Levels of Inflammation Biomarkers from Depression Risk Scores Calculated at Age 12.

Inflammation Biomarker	Model	N	Coefficient	95% CI	*p*
CRP level (mg/L)	1	1,026	**2.52**	**0.83 – 4.21**	**0.004**
	2	981	0.32	−1.76 – 2.41	0.760
IL-6 level (pg/mL)	1	1,034	0.13	−0.43 – 0.70	0.646
	2	989	−0.10	−0.85 – 0.65	0.786
suPAR level (ng/mL)	1	1,039	**3.95**	**2.87 – 5.03**	**<0.001**
	2	994	**1.70**	**0.46 – 2.95**	**0.007**

*Note*. CI = confidence interval; CRP = C-reactive protein; IL-6 = interleukin-6; suPAR = soluble urokinase plasminogen activator receptor. Model 1 is adjusted for familial clustering only. Model 2 is adjusted for familial clustering, body mass index, body temperature, smoking, sex, and prior depression. Statistically significant (*p* < 0.05) associations are shown in bold.

Table 2 shows the associations between MDD risk group membership (low- versus high-risk) and levels of each inflammation biomarker at age 18. MDD risk group membership was not associated with subsequent levels of CRP or IL-6. However, being at high risk of developing MDD was associated with significantly higher subsequent levels of suPAR compared with being at low risk of MDD. This association remained when models were adjusted for BMI, body temperature, smoking, and previous depression.

**Table 2:** Regression Models Predicting Age-18 Levels of Inflammation Biomarkers from Age-12 Depression Risk Group Membership (low- versus high-risk).

Inflammation Biomarker	Model	Coefficient	95% CI	*p*
CRP level (mg/L)	1	0.34	−0.03 – 0.70	0.070
	2	0.29	−0.06 – 0.65	0.103
IL-6 level (pg/mL)	1	−0.01	−0.16 – 0.14	0.887
	2	0.02	−0.14 – 0.18	0.815
suPAR level (ng/mL)	1	**0.51**	**0.28 – 0.75**	**<0.001**
	2	**0.41**	**0.18 – 0.64**	**<0.001**

*Note*. CI = confidence interval; CRP = C-reactive protein; IL-6 = interleukin-6; suPAR = soluble urokinase plasminogen activator receptor. Model 1 is adjusted for familial clustering only (N = 287). Model 2 is adjusted for familial clustering, body mass index, body temperature, smoking, and prior depression (N = 273). Low-risk group defined as a risk score for age-18 MDD ≤ 10th percentile; high-risk group defined as a risk score for age-18 MDD ≥ 90th percentile. Statistically significant (*p* < 0.05) associations are shown in bold.

## 4 Discussion

Using a UK nationally representative cohort, we investigated associations between individual risk for developing future MDD and subsequent levels of inflammation biomarkers. Our results showed that risk at age 12 for developing MDD at age 18 was positively associated with age-18 levels of suPAR. Adolescents who were identified as being at high risk for developing MDD (in the top 90th percentile) subsequently had significantly higher levels of suPAR compared to those who had been identified as being at low risk (in the lowest 10th percentile). No association was found between individual MDD risk and subsequent levels of CRP or IL-6 after adjusting for key covariates.

Our finding that levels of suPAR were elevated among those who were previously identified as being at high risk for developing MDD is consistent with literature suggesting that inflammation is involved in the development of depression (Slavich and Irwin, 2014). However, unlike some previous studies that have found elevated levels of CRP and IL-6 to be linked with depression (Howren et al., 2009, Khandaker et al., 2014, Zalli et al., 2016), individual risk for MDD was unrelated to these two biomarkers in our study. That risk for MDD was associated with suPAR but not CRP or IL-6 may be understood as reflecting an enduring inflammatory response rather than acute fluctuations (Rasmussen et al., 2021a, Rasmussen et al., 2021b). CRP and IL-6 are involved in the acute-phase response and sensitive to short-term influences such as infections (Hunter and Jones, 2015, Rhodes et al., 2011) and thus may mix chronic and acute effects such that levels of these biomarkers within the body can fluctuate over time. Given that our measurement of inflammation was taken six years after individual MDD risk was assessed this may account for why we found no association with these inflammatory markers. In contrast, suPAR is considered to be a more stable inflammatory marker that could better index systemic chronic inflammation (Rasmussen et al., 2021a, Rasmussen et al., 2021b) and therefore it may be more appropriate for capturing biologically embedded risk over a longer period of time.

By demonstrating that individual risk for MDD arising from a combination of multiple sociodemographic sources is associated with inflammation in the form of elevated levels of suPAR six years later, our study supports and extends existing research on links between childhood adversity and inflammation (Baldwin et al., 2018, Danese et al., 2007, Giletta et al., 2018, Rasmussen et al., 2020). It is likely that our findings, in part, reflect the inclusion of childhood maltreatment as one of the predictors in the risk prediction model. Furthermore, it is possible that other model predictors (e.g., history of running away from home, drug use) may partially capture aspects of childhood adversity. However, our study draws on a wider range of risk factors for MDD than has previously been examined in relation to inflammation and uses more sophisticated modelling techniques to combine these into individual-level MDD risk scores.

Targeted intervention for people who have been identified as being at high risk for developing MDD is critical for preventive efforts and is likely to be more effective than universal approaches (Hetrick et al., 2015). Furthermore, identifying such high-risk individuals before they become unwell may be preferable to intervening in response to mental ill-health, at which point successful treatment can be challenging (Rush et al., 2006). Because of their elevated levels of suPAR, individuals identified as being at high risk for depression may also be vulnerable to other problematic conditions such as cardiovascular disease and diabetes (Eugen-Olsen et al., 2010), and at increased risk of accelerated biological aging (Rasmussen et al., 2021a) and mortality (Rasmussen et al., 2016). Therefore, interventions to reduce suPAR levels among those at high-risk for MDD may prevent not only future depression but a range of adverse physical health outcomes too. Such preventive interventions need not involve medication. Indeed, a recent study found that having an unhealthy lifestyle was associated with increases in suPAR levels over time (Haupt et al., 2019) and thus changes in lifestyle habits may be useful and less intrusive preventive interventions.

## 5 Limitations

Our findings should be considered in context of this study’s limitations. First, inflammation was measured only at age 18 and therefore we were unable to control for potentially elevated inflammatory levels at earlier time-points or explore how levels changed over time. Second, blood plasma samples were not available for all participants in the E-Risk study. However, there were no differences in MDD risk score for those with and without inflammation data available. Third, we used a twin sample and the extent to which findings from twins generalise to non-twins is sometimes questioned. However, the prevalence of mental health problems for twins and non-twins has been found to be comparable (Kendler, et al., 1995) and the E-Risk sample is representative of UK families in terms of geographical and socio-economic distribution (Odgers et al., 2012).

### 5.1 Conclusion

Individual risk for developing MDD at age 18, calculated using a multivariable prognostic model at age 12, was associated with levels of suPAR (but not CRP or IL-6) measured at age 18. Adolescents who had high MDD risk scores had significantly higher subsequent levels of suPAR than those who had low risk scores. Findings extend the limited literature on links between MDD risk factors and inflammation in adolescence and young adulthood and support the notion that childhood psychosocial risk for MDD becomes biologically embedded. If our findings are replicated in larger samples with assessment of inflammation biomarkers at multiple points throughout childhood and adolescence, then they may support the use of targeted interventions in adolescents at high-risk for MDD to reduce suPAR levels. This in turn may prevent the development of depression as well as major physical health problems and mortality.

## 6 Role of the funding source

The E-Risk Study is funded by the Medical Research Council (UK MRC) [G1002190]. Additional support was provided by the US National Institute of Child Health and Human Development (NICHD) [HD077482]; the Jacobs Foundation, Switzerland; the King’s Together Multi and Interdisciplinary Research Scheme (UK Wellcome Trust Institutional Strategic Support Fund [204823/Z/16/Z]); UK MQ Transforming Mental Health Charity, Brighter Futures grant named “Identifying Depression Early in Adolescence” [MQBF/1 IDEA]; plus the UK MRC [MC_PC_MR/R019460/1] and the UK Academy of Medical Sciences [GCRFNG\100281] under the Global Challenges Research Fund. Helen L. Fisher and Rachel M. Latham are supported by the UK Economic and Social Research Council (ESRC) Centre for Society and Mental Health at King’s College London [ES/S012567/1]. Louise Arseneault is the Mental Health Leadership Fellow for the UK ESRC. Valeria Mondelli was part funded by the UK National Institute for Health Research (NIHR) Biomedical Research Centre at South London and Maudsley NHS Foundation Trust and King’s College London. Christian Kieling has received support from Brazilian governmental research funding agencies (Conselho Nacional de Desenvolvimento Científico e Tecnológico [477129/2012-9 and 445828/2014-5], Coordenação de Aperfeiçoamento de Pessoal de Nível Superior [62/2014], and Fundação de Amparo à Pesquisa do Estado do Rio Grande do Sul [17/2551-0001009-4], Brazil) and is a UK Academy of Medical Sciences Newton Advanced Fellow. Brandon Kohrt has received support from the US National Institute of Mental Health (NIMH) [R01MH120649]. Line J. H. Rasmussen is supported by an international postdoctoral fellowship from the Lundbeck Foundation in Denmark [R288-2018-380]. The views expressed are those of the authors and not necessarily those of the National Health Service, the NIHR, the Department of Health and Social Care, the ESRC or King’s College London. These funders played no role in study design; in the collection, analysis and interpretation of data; in the writing of the report; nor in the decision to submit this article for publication.

## Declaration of Competing Interest

The authors declare the following financial interests/personal relationships which may be considered as potential competing interests: Valeria Mondelli has received research funding from Johnson & Johnson as part of a research program on depression and inflammation, but the research described in this paper is unrelated to this funding. All other authors declare they have no conflicts of interest to report.
