# Supplementary material for: Longitudinal associations between adolescents’ individualised risk for depression and inflammation in a UK cohort study
Source: Brain Behav Immun. 2022 Mar;101:78–83. doi: 10.1016/j.bbi.2021.12.027 (PMC8906711; doi:10.1016/j.bbi.2021.12.027)
Supplement: Supplementary data 1 [file mmc1.docx]

**Supplementary Material**

**Methods**

**Study Cohort**

Participants were members of the Environmental Risk (E-Risk) Longitudinal Twin Study, which tracks the development of a nationally representative birth cohort of 2,232 British twin children. The sample was drawn from a larger birth register of twins born in England and Wales in 1994-1995 (Trouton, Spinath, and Plomin, 2002). Full details about the sample are reported elsewhere (Moffitt and E-Risk Study Team, 2002). Briefly, the E-Risk sample was constructed in 1999-2000, when 1,116 families (93% of those eligible) with same-sex 5-year-old twins participated in home-visit assessments. This sample comprised 56% monozygotic (MZ) and 44% dizygotic (DZ) twin pairs; sex was evenly distributed within zygosity (49% male). Families were recruited to represent the UK population of families with newborns in the 1990s, on the basis of residential location throughout England and Wales and mother’s age. Teenaged mothers with twins were over-selected to replace high-risk families who were selectively lost to the register through non-response. Older mothers having twins via assisted reproduction were under-selected to avoid an excess of well-educated older mothers.

Follow-up home-visits were conducted when children were aged 7, 10, 12 and 18 (participation rates were 98%, 96%, 96% and 93%, respectively). Home-visits at ages 5, 7, 10, and 12 years included assessments with participants as well as their mother (or primary caregiver); the home-visit at age 18 included interviews only with the participants. Each participant in a twin pair was assessed by a different interviewer. There were 2,066 E-Risk participants who were assessed at age 18. The average age of the participants at the time of the assessment was 18.4 years (SD = 0.36); all interviews were conducted after the 18th birthday. There were no differences between those who did and did not take part at age 18 in terms of socioeconomic status (SES) assessed when the cohort was initially defined (χ^2^ = 0.86, p = 0.65), age-5 IQ scores (t = 0.98, p = 0.33), age-5 internalising or externalising behaviour problems (t = 0.40, p = 0.69 and t = 0.41, p = 0.68, respectively), or childhood poly-victimisation (z=0.51, p=0.61). The study sample represents the full range of socioeconomic conditions in Great Britain, as reflected in the families’ distribution on a neighbourhood-level socioeconomic index (ACORN [A Classification of Residential Neighbourhoods] developed by CACI Inc. for commercial use; CACI Information Services, 2006). E-Risk families ACORN distribution closely matches that of households nation-wide: 25.6% of E-Risk families live in “wealthy achiever” neighbourhoods compared to 25.3% of households nation-wide; 5.3% vs 11.6% live in “urban prosperity” neighbourhoods; 29.6% vs 26.9% live in “comfortably off” neighbourhoods; 13.4% vs 13.9% live in “moderate means” neighbourhoods; and 26.1% vs 20.7% live in “hard-pressed” neighbourhoods. E-Risk underrepresents urban prosperity neighbourhoods because such households are likely to be childless.

Parents gave informed consent and twins gave assent between 5-12 years and then informed consent at age 18. The Joint South London and Maudsley and the Institute of Psychiatry Research Ethics Committee approved each phase of the study.

***Supplementary Figure S1.*** The E-Risk Study families’ addresses are a near-perfect match to the deciles of the UK government’s Index of Multiple Deprivation.

*Note.* This histogram shows E-Risk families’ addresses are a near-perfect match to the deciles of the UK’s 2015 Lower-layer Super Output Area (LSOA) Index of Multiple Deprivation (IMD) which averages 1,500 residents (or 650 households each); approximately 10% (dotted red line) of the E-Risk cohort fills each of the IMD’s 10% bands, indicating that the E-Risk cohort accurately represents the distribution of deprivation in the UK.

**MDD Risk Prediction Model**

The development and external validation of the MDD risk prediction model are described in full by Rocha et al. (2021). In brief, data from the 1993 Pelotas Birth Cohort were used to develop the prediction model, and its generalisability was then evaluated in the E-Risk Longitudinal Twin Study. As variables available in the E-Risk Study dataset did not perfectly pair with those selected from the Pelotas study, the Pelotas model was rebuilt, and the linear predictor recalculated considering only the variables available for comparison (i.e., biological sex, skin colour, any drug use, school failure, social isolation, fight involvement, ever ran away from home, childhood maltreatment, and interactions of each of these with biological sex). Consistent with methodological recommendations (Moons et al., 2012) the model intercept was corrected for the E-Risk cohort to take account of the differing MDD prevalence rates (model recalibration). Model coefficients were also optimised for the E-Risk cohort to account for differences in the strength of predictors. Performance statistics for this refitted model (as reported by Rocha et al., 2021) are shown in Supplementary Table S1.

**Supplementary Table S1.** Model Performance of the MDD Risk Prediction Model Refitted to the E-Risk Cohort (as reported in Table 3 of Rocha et al., 2021)

| **Performance measure** | **Description** | **E-Risk refitted model** |
| --- | --- | --- |
| C-Statistic | Measure of discrimination – the model’s ability to distinguish between those with and without depression. It can vary between 0.5 and 1 and is equal to the area under the curve of the receiver operating characteristic (AUC-ROC) for binary outcomes | 0.62 |
| Calibration intercept | An overall measure of calibration – compares mean observed with mean predicted | 0.00 |
| Calibration slope | Measure of agreement between observed and predicted risk of the outcome across the whole range of predicted values | 1.20 |
| R^2^ | Measure of overall goodness-of-fit of the model | 0.05 |
| Brier Score | Quadratic scoring rule that combines calibration and discrimination | 0.14 |

*Note*. Better model performance is indicated by higher values for C-statistic and R^2^, lower values for Brier score, values closer to 0 for calibration intercept, and values closer to 1 for calibration slope.

**Supplementary Table S2.** Definition and Measurement of Variables Used for the MDD Risk Prediction Model Externally Validated Using the E-Risk Cohort (Rocha et al., 2021).

| **Variable (reference category)^a^** | **Measurement in E-Risk cohort** |
| --- | --- |
| Previous depression screening^b^ | Any evidence (“OR” rule) of depressive symptoms, assessed at ages 5, 7 and 10 by a depression subscale derived from a combination of mother and teacher CBCL for emotional problems, using the 93^rd^ percentile as cut-point, and at age 12 by self-reported CDI scores (with a clinical cut-off >= 20) |
| Sex (male)^c^ | Self-reported sex |
| Skin colour (white)^c^ | Parent-reported ethnicity |
| Childhood maltreatment (None)^c^ | Prospectively obtained variable for sexual/physical abuse up to age 12 based on mother reports, researcher observations, and social services referral information, coded as none,  probable, or definite; inserted as a categorical variable into the model |
| School failure (No)^c^ | Evaluation of sample’s distribution of English/Math performance at age 12, considering those below the 20^th^ percentile as “failing at school”=1; otherwise=0 |
| Social Isolation (0)^c^ | Combination of CBCL and TRF items on social isolation was pooled into a 3 strata categorical social isolation variable (low, moderate and high social isolation; Matthews et al., 2015), and then reclassified into a dichotomised variable: high social isolation=1 and low/moderate=0 |
| Fights (No)^c^ | Combination (“AND” rule) of two dichotomous questions: “Do you sometimes hit someone when you are having an argument?” and “Do you sometimes start fights with people?”; positive answers to both questions were classified as “1”; otherwise “0” |
| Ran away (No)^c^ | Responses to the dichotomous question: “Have you run away from home and stayed away for the night?”; positive answers were classified as “1”, and negative as “0” |
| Drug use (No)^c^ | Dichotomous variable combining responses to dichotomous questions about any lifetime use of alcohol, tobacco, cannabis, pills and inhalants; any positive answer=“1”; otherwise=“0” |
| Depression diagnosis at age 18^d^ | Evaluation of major depressive episode diagnosis with DSM-IV criteria in the previous 12 months |

*Note*. ^a^For variables included as predictors. ^b^Those with prior depressive symptoms were excluded. ^c^Predictor variable in the MDD risk prediction model. ^d^Outcome variable for the MDD risk prediction model. CBCL=Child Behaviour Checklist; CDI=Children’s Depression Inventory; DSM = Diagnostic and Statistical Manual of Mental Disorders; E-Risk=Environmental Risk Longitudinal Twin Study; TRF=Teacher’s Report Form.

**References**

CACI Information Services (2006). ACORN User Guide. London, UK: CACI

Matthews, T., Danese, A., Wertz, J., Ambler, A., Kelly, M., Diver, A., Caspi, A., Moffitt, T.E., Arseneault, L., 2015. Social isolation and mental health at primary and secondary school entry: a longitudinal cohort study. JAMA Psychiatry, 54, 225-32. https://doi.org/10.1016/j.jaac.2014.12.008.

Moffitt, T.E., the E-Risk Study Team, 2002. Teen-aged mothers in contemporary Britain. J. Child Psychol. Psychiatry. 43, 727–742. https://doi.org/[10.1111/1469-7610.00082](https://doi.org/10.1111/1469-7610.00082)

Moons, K.G., Kengne, A.P., Grobbee, D.E., Royston, P., Vergouwe, Y., Altman, D.G., Woodward, M., 2012. Risk prediction models: II. External validation, model updating, and impact assessment. Heart, 98, 691–698. https://doi.org/10.1136/heartjnl-2011-301247

Rocha, T.B.M., Fisher, H.L., Caye, A., Anselmi, L., Arseneault, L., Barros, F.C., Caspi, A., Danese, A., Gonçalves, H., Harrington, H., Houtes, R., Menezes, A.M.B., Moffitt, T.E., Mondelli, V., Poulton, R., Rohde, L.A., Wehrmeister, F., Kieling, C., 2021. Identifying adolescents at risk for depression: a prediction score performance in cohorts based in three different continents. J. Am. Acad. Child Adolesc. Psychiatry, 60, 262–273. <https://doi.org/10.1016/j.jaac.2019.12.004>

Trouton, A., Spinath, F. M., Plomin, R., 2002. Twins early development study (TEDS): A multivariate, longitudinal genetic investigation of language, cognition and behavior problems in childhood. Twin Res. Hum. Genet. 5, 444-448. <https://doi.org/10.1375/twin.5.5.444>
